# Health and productivity benefits of a multicomponent workplace program by gender in small companies

**DOI:** 10.3389/fspor.2025.1672619

**Published:** 2025-08-29

**Authors:** Víctor Jiménez Díaz-Benito, Jose Bonal, Alvaro Fernandez-Luna, Pablo Burillo, Ricardo Macías, Jairo León-Quismondo

**Affiliations:** ^1^ Department of Sports Sciences, Faculty of Medicine, Health and Sports, Universidad Europea de Madrid, Madrid, Spain; ^2^ Department of Real Madrid Graduate School, Faculty of Medicine, Health and Sports, Universidad Europea de Madrid, Madrid, Spain

**Keywords:** workplace, sustainable employability, productivity, management, exercise, program, women

## Abstract

**Background:**

Physical inactivity is a major public health concern, with persistent gender disparities and growing sedentary behavior in modern workplaces. Small- and medium-sized enterprises (SMEs), despite employing most of the workforce, often lack structured health programs. Workplace physical activity interventions show promise but yield mixed results, especially when gender differences are not considered. This study addresses these gaps by evaluating a supervised exercise program through a sex-disaggregated analysis in SMEs from diverse sectors.

**Methods:**

We conducted a 12-week multicomponent physical activity program in three SMEs, using a mixed factorial design to assess changes by time, gender, and company. Physically inactive employees with sedentary jobs participated in supervised exercise sessions and health-promotion activities. The study included employees from three sectors: Training and Consulting, Insurance and Technology, and Digital Engineering, with a total sample of 49 participants (41 women and 8 men). Pre-and post-intervention measures included anthropometrics, perceived health, cardiorespiratory fitness, and productivity.

**Results:**

Cardiovascular fitness improved significantly in both sexes, and diastolic blood pressure decreased over time across companies. Quality-of-life scores did not improve globally. Productivity costs due to presenteeism decreased significantly, with *post hoc* differences by company and sex (*p* < .001).

**Discussion:**

A tailored three-month supervised exercise program, delivered onsite or online three times per week, appears to improve health outcomes among SME administrative staff and reduce productivity losses, especially when combining aerobic and strength training with activation routines, workshops, and gamified challenges.

## Introduction

Physical inactivity is a well-established risk factor for chronic diseases and reduced well-being (1). Physical inactivity is now recognised as a global pandemic: estimates from the World Health Organization's estimates, a third of adults (around 1.8 billion people) fail to achieve the minimum 150 min week of moderate-intensity activity, with women remaining around 5% less active than men, a disparity unchanged since 2000 (2). Beyond avoidable morbidity and mortality, insufficient physical activity imposes a substantial economic burden. Previous analyses calculate that closing the inactivity gap across the European Union would save health-care systems nearly €8 billion annually and avoid further productivity losses (3).

**Table 1:** Sample characteristics.

Outcome	Gender	Training and consulting	Gender	Insurance and technology	Gender	Digital engineering
Age (years)	Men (*n* = 5)	42.80 (11.23)	Men (*n* = 4)	27.25 (19.92)	Men (*n* = 4)	29.25 (6.34)
	Women (*n* = 32)	33.81 (9.27)	Women (*n* = 0)	n/d	Women (*n* = 1)	27.00 (0.00)
Weight (kg)	Men (*n* = 7)	79.88 (6.52)	Men (*n* = 4)	83.72 (3.38)	Men (*n* = 4)	93.30 (18.35)
	Women (*n* = 33)	68.56 (17.09)	Women (*n* = 0)	n/d	Women (*n* = 1)	73.40 (0.00)
Body mass index (BMI)	Men (*n* = 7)	25.21 (1.27)	Men (*n* = 4)	26.23 (2.21)	Men (*n* = 4)	31.10 (5.82)
	Women (*n* = 33)	24.57 (7.22)	Women (*n* = 0)	n/d	Women (*n* = 1)	27.90 (0.00)
Body fat (%)	Men (*n* = 7)	24.61 (2.79)	Men (*n* = 4)	24.35 (4.76)	Men (*n* = 4)	31.28 (9.99)
	Women (*n* = 33)	30.07 (8.62)	Women (*n* = 0)	n/d	Women (*n* = 1)	32.10 (0.00)

Mean (SD); *n*/d, no data.

Because adults spend about one-third of their waking hours at work, workplaces have emerged as a fundamental setting for large-scale health promotion (4). Within this broader context of global inactivity, the occupational environment has undergone a parallel transformation, shifting from physically demanding roles to predominantly sedentary ones (5). This transformation reinforces the need to reconsider how workplaces can actively contribute to public health efforts.

The proportion of physically active occupations has dropped to fewer than 20% today (1). As modern work environments have grown increasingly sedentary, the workplace has become a crucial setting for promoting regular exercise. In response, many employers have implemented workplace physical activity programs (WPPAs) to improve employee health and productivity. International health strategies encourage such initiatives as part of broader efforts to combat non-communicable diseases and achieve sustainable development goals (6, 7).

Small- and medium-sized enterprises (SMEs) deserve special attention. They account for 99% of all EU businesses and provide two-thirds of private-sector employment (8), yet often lack the occupational-health infrastructure available in larger corporations (9). Responding to this unmet need, WHO Europe (10) published a dedicated guide urging SMEs to implement gender-responsive physical-activity policies. In Spain, where SMEs account for 64.5% of jobs (11), international health monitoring still reports particularly high sedentary behaviour, and chronic health problems among women, underscoring the urgency of context-specific interventions (2, 12).

Research to date indicates that WPPAs can produce meaningful benefits for both workers and organizations. Well-designed workplace physical activity programs that are multicomponent and tailored to the specific demands of each job type have demonstrated promising outcomes across health-related predictors such as blood pressure, cardiovascular function, and metabolic markers (13–17). Recent systematic reviews report consistent improvements in employees' physical health indicators, including cardiorespiratory fitness and muscular strength, following workplace exercise interventions (7).

Positive impacts on work-related outcomes, such as enhanced work ability and reduced absenteeism, have also been documented (7). Furthermore, many interventions show gains in psychosocial well-being. For example, in previous research, over 60% of trials found reductions in employees' stress levels after participating in workplace exercise programs (18). Nevertheless, effect sizes are often modest and variable, suggesting that program effectiveness may depend on context and participant characteristics.

These findings raise the possibility that the scientific evidence base may be equivocal. The influential meta-analysis by Conn et al. (13) documented highly variable effects of workplace programmes on cardiorespiratory fitness, body composition and productivity. Recent evidence reflects similar heterogeneity: a 2022 systematic review of 39 studies reported effective interventions in only 41% of randomised trials and highlighted inconsistent reporting of implementation details (19); another review of 36 systematic reviews found only small improvements in daily steps and no convincing cardiometabolic benefits, while rating 78% of reviews as low quality (20). Digital-health interventions are considered suitable for large-scale implementation. However, previous research suggests that e-workplace tools provide limited additional benefit over traditional programmes and may even widen equity gaps, as engagement often differs by sex, age, and socioeconomic background (21). In this regard, the information received by participants has proven to be an important factor (22).

One important source of variability is gender. Globally, women tend to be less physically active than men (32% of women are insufficiently active, compared to 23% of men), reflecting a persistent gender gap in activity levels (1, 23). Women also face distinct barriers to exercise: they more frequently cite lack of time, energy, and social support as obstacles (24). Interestingly, survey data indicate that, despite higher overall activity levels, male employees are significantly less likely than females to participate in worksite health programs (25, 26). Cultural factors likely contribute to these patterns: women are often more health-conscious and engage in health-promoting behaviors, whereas prevailing gender roles that emphasize independence in men may discourage some from participating in wellness activities (26). These gender differences in participation and behavior could lead to differential outcomes in workplace interventions.

Despite this, relatively few studies have explicitly examined sex differences in the impact of workplace physical activity interventions (27). Most evaluations aggregate results for both sexes or focus on a predominately single-gender sample, leaving it unclear whether men and women benefit equally from the same program (28). This represents a notable gap in the literature, especially given calls for more gender-sensitive approaches in workplace health promotion (26). Moreover, the need for sex-disaggregated evidence is particularly pronounced in small and medium-sized enterprises (SMEs), which employ a large segment of the workforce yet are understudied in intervention research. The processes of building scientific evidence on interventions from a gender perspective go beyond purely clinical reasoning; they represent a significant multidisciplinary scientific movement towards social inclusion (29). Addressing these gaps would ensure that workplace wellness strategies are effective and equitable for all employees.

Considering these gaps in the literature, the present study aimed to evaluate the effects of a 12-week supervised physical activity program in the workplace on employees' health, perceived well-being, and productivity, with a specific focus on comparing outcomes between male and female workers. This sex-disaggregated evaluation seeks to determine whether a standardized exercise intervention yields similar benefits for men and women, thereby contributing new insights into gender-specific impacts of workplace health programs.

## Methods

### Design

Were utilized a mixed factorial experimental design to assess the impact of a workplace exercise program on participants’ health and productivity. The design included two between-subjects factors: workplace group (three small companies) and gender (male and female), as well as one within-subjects factor: time (pre-intervention and post-intervention). This allowed for the analysis of main effects and interactions between group, gender, and time, to evaluate whether the changes observed from pre- to post-intervention differed according to the workplace and the sex of the participants.

### Participants

**Figure 1:**
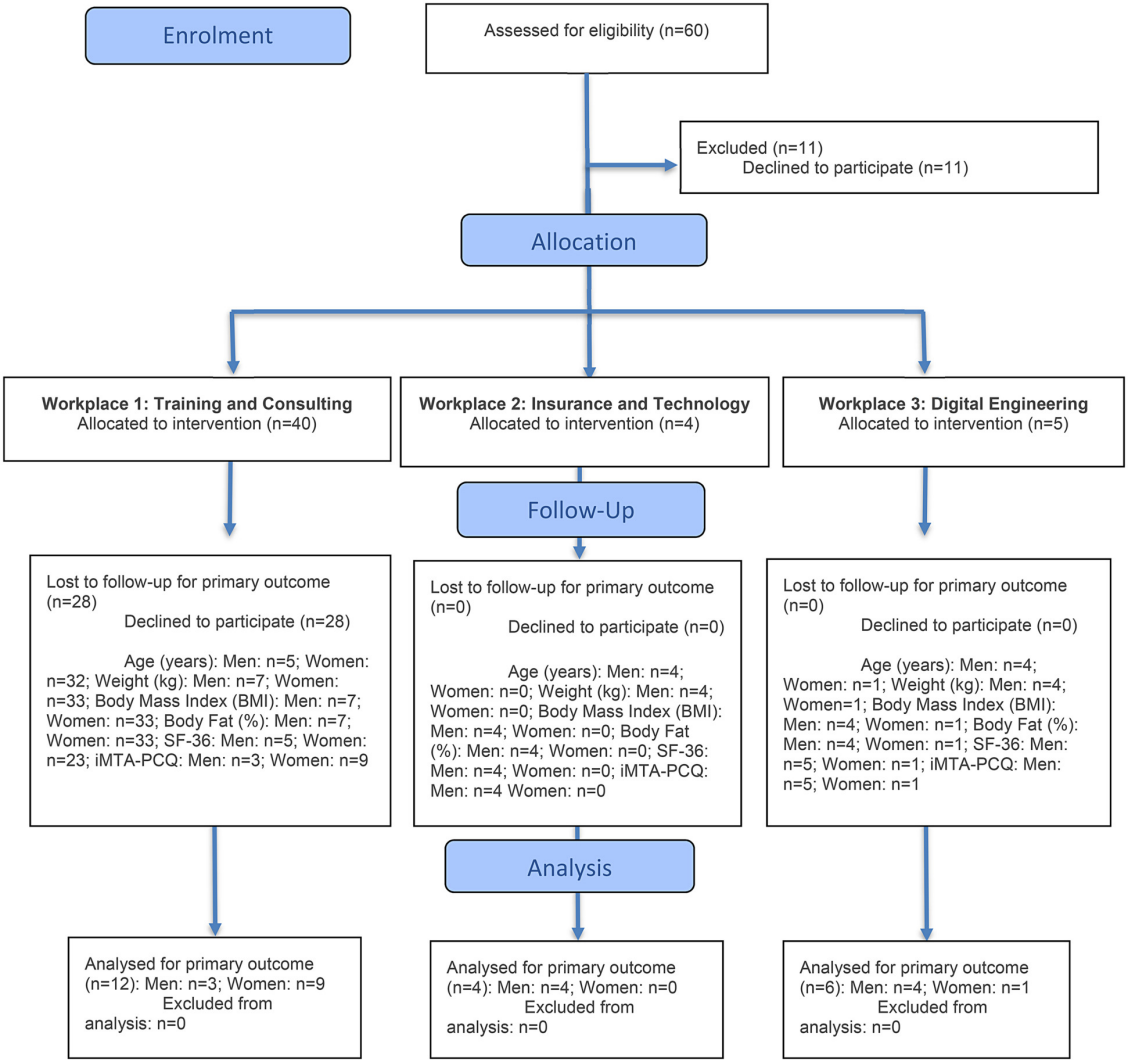
CONSORT 2025 flow diagram.

A convenience sampling method was used for participant selection. Recruitment was carried out through internal communication channels, primarily via email. To be eligible for the study, participants had to meet the following inclusion criteria. Be over 16 years of age, have a full-time employment contract with the company, and be physically inactive. Physical inactivity levels were assessed through a personal interview conducted by a PhD in Physical Activity and Sport Sciences, who is also a licensed Physical Activity and Sport Educator. Exclusion criteria included being pregnant, having medical contraindications for performing supervised physical exercise, not having a sedentary office-based job, or being physically active with regular and ongoing access to physical activity. All participants provided written informed consent prior to participation. They were recruited from three small to medium-sized enterprises (SMEs) from different sectors: a training and consulting company, an insurance and technology company, and a digital engineering company. All data were processed voluntarily and anonymously, and recorded in a secure database accessible only to the principal investigator. The study was conducted in accordance with the Declaration of Helsinki (30), the guidelines of the Ethics Committee, and the applicable national data protection legislation. A flow diagram detailing participant inclusion is provided below (Figure 1).

### Intervention

The physical exercise program was designed by a PhD in Physical Activity and Sport Sciences and delivered by three licensed Physical Activity and Sport Educators (graduates in Physical Activity and Sport Sciences). The intervention lasted 12 weeks, from April to July, with three 60-minute sessions per week. Sessions were held outside regular working hours, scheduled in various time slots and organized into groups based on each participant's prior availability and preferences, as previously reported. Participants could choose between online sessions or in-person sessions, either at the office or in a nearby park, with all details communicated in advance through internal channels. To ensure external validity, switching groups was not permitted once the program had begun. Sessions began at an intensity of 55%–60% of the participants' maximum heart rate (HRmax), progressively increasing to 75%–80%. Exercise intensity was prescribed using the Karvonen formula. Physical activity levels and intensity were monitored through self-reported activity logs and heart rate tracking. Before the program began, participants received a participant guide, were assigned anonymized codes, and completed initial questionnaires and physical fitness assessments. They also attended a training session based on the World Health Organization's physical activity recommendations. The program was individually adapted to meet the participants' needs, characteristics, and expectations, prescribing the appropriate type and dosage of exercise for each worker. The program was complemented by the Workplace Fitness & Nutrition component, which included: (I) Nutritional guidelines; (II) Healthy recipes; (III) More than 30 on-demand workout sessions led by three different trainers; and (IV) Gamified challenges and newsletters. The intervention was delivered in both in-person and online formats and included the following components: (I) Concurrent and supervised resistance and strength training targeting all major muscle groups (50 min, 3 times per week); (II) Morning activation sessions focusing on joint mobility and core, back, neck, and upper limb training (15 min, 5 times per week); (III) Gamified challenges (3 in total); (IV) Health promotion and education program focused on physical activity and nutrition; and (V) Newsletters with tips, health content, challenges, and more (8 issues). The entire program followed a quarterly planning structure, with specific content organized by activity block.

### Measurements

To evaluate the impact of the program, demographic, clinical, and self-reported data were collected through personal interviews and anthropometric measurements. Anthropometric measures included weight (kg) and Body Mass Index (BMI), calculated as weight/height^2^ (kg/m^2^), using a TANITA HD-383 scale. Body fat percentage (%) was estimated using the formula proposed by Deurenberg et al. (42). Perceived health and quality of life were assessed using the SF-12v2 Health Survey (0–100 scale) (31). Productivity losses due to health-related issues, including both absenteeism and presenteeism, were measured using the iMTA Productivity Cost Questionnaire (32). General physical fitness and cardiorespiratory recovery capacity were evaluated through the Ruffier-Dickson Test (33).

### Analyses

The analyses were performed using the IBM SPSS 29.0 statistical software package (IBM Corp., Armonk, NY, USA). Initially, goodness-of-fit tests were conducted using the Kolmogorov–Smirnov technique (*N* > 30). Multivariable analysis was carried out through multivariate analysis of variance (ANOVA). *post hoc* multiple comparisons were performed using the Bonferroni method. Effect size was calculated using Eta squared (*η*^2^) (*η*^2^ = Z^2^/N, where N is the number of observations), with values of 0.01, 0.06, and 0.14 interpreted as small, medium, and large effect sizes, respectively (43). We first assessed the assumption of sphericity. When Mauchly's test yielded a value greater than 0.05, results were interpreted using the Huynh-Feldt correction. For multivariate significance testing, Pillai's trace was used (44). The significance level was set at 0.05 for all tests.

## Results

### Sample description

The study sample (Table 1) comprised employees from three different sectors: Training and Consulting, Insurance and Technology, and Digital Engineering. In the Training and Consulting sector, there were 39 participants (7 men and 32 women). The average age was 42.8 years (SD = 11.23) for men and 33.8 years (SD = 9.27) for women. Men had an average weight of 79.9 kg (SD = 6.52), a mean BMI of 25.2 (SD = 1.27), and an average body fat percentage of 24.6% (SD = 2.79). Women in this sector averaged 68.6 kg (SD = 17.1) in weight, a BMI of 24.6 (SD = 7.22), and 30.1% (SD = 8.62) body fat. The Insurance and Technology sector included only male participants (*n* = 4), with a mean age of 27.3 years (SD = 19.92), an average weight of 83.7 kg (SD = 3.38), BMI of 26.2 (SD = 2.21), and body fat percentage of 24.4% (SD = 4.76). The Digital Engineering sector had five participants (4 men and 1 woman). Men had a mean age of 29.3 years (SD = 6.34), weight of 93.3 kg (SD = 18.35), BMI of 31.1 (SD = 5.82), and body fat percentage of 31.3% (SD = 9.99). The single female participant was 27 years old, weighed 73.4 kg, had a BMI of 27.9, and body fat of 32.1%. Concerning educational attainment, more than half of the participants (52.5%) had completed university studies. Program adherence exceeded 60%. Additionally, 11.9% had completed higher vocational training, 1.7% held a high school diploma, 5.1% completed intermediate vocational training, 6.8% had secondary education, and 5.1% reported other qualifications.

### Physical and anthropometric measures

A significant decrease in diastolic blood pressure was found over time across the different companies (F₁=6.55; *p* = .021; *η*^2^ₚ=.291). In *post hoc* multiple comparisons, statistically significant differences in cardiovascular fitness were found between the companies engaged in insurance and technology and digital engineering (*p* = .033). No statistically significant differences were observed by sex across the different companies. Diastolic blood pressure decreased by around 5% in both sexes, but we didn't found statically differencies (*p* = .09). Cardiovascular fitness showed a statistically significant improvement from pre- to postintervention in both sexes (see Table 2), with improvements close to 30% in both groups (*p* = .003).

**Table 2:** Descriptive statistics and repeated measures ANOVA by gender and workplace (pre and post-intervention).

Outcome	Gender	Company	N	Pre M	Pre SD	Post M	Post SD	F	*p*	*η*^2^p
Weight (kg)	Men	Insurance and technology	4	83.73	3.38	83.05	4.99	.16	.691	
		Digital engineering	3	97.13	20.43	98.17	24.28			
	Women	Training and consulting	12	74.63	16.01	74.49	16.87			
		Digital engineering	1	73.40	.00	74.80	.00			
Body mass index (BMI)	Men	Insurance and technology	4	26.23	2.21	26.10	2.58	.55	.470	
		Digital engineering	3	31.93	6.83	32.37	8.02			
	Women	Training and consulting	12	26.88	5.84	26.78	6.09			
		Digital engineering	1	27.90	n/d	28.50	n/d			
Body fat (%)	Men	Insurance and technology	4	24.35	4.76	24.63	5.06	.22	.640	
		Digital engineering	3	32.80	11.65	33.53	13.36			
	Women	Training and consulting	12	32.29	8.93	31.37	9.47			
		Digital engineering	1	32.10	.00	31.20	.00			
Systolic BP (mm Hg)	Men	Insurance and technology	4	122.50	32.70	118.75	13.40	2.04	.164	
		Digital engineering	3	117.33	15.01	116.00	14.73			
	Women	Training and consulting	12	117.14	11.99	112.58	7.81			
		Digital engineering	1	118.00	.00	129.00	.00			
Diastolic BP (mm Hg)	Men	Insurance and technology	4	74.75	19.16	68.25	18.30	6.55	.021^*^	**.291**
		Digital engineering	3	78.67	17.21	72.33	12.66			
	Women	Training and consulting	12	72.58	6.19	69.75	4.88			
		Digital engineering	1	69.00	.00	65.00	.00			
Ruffier-Dickson Index	Men	Insurance and technology	4	7.90	4.25	6.93	3.85	1.50	.230	
		Digital engineering	3	16.00	4.16	12.27	8.72			
	Women	Training and consulting	12	12.53	4.16	10.21	4.05			
		Digital engineering	1	18.10	.00	19.00	.00			

Bold values highlight statistically significant differences (*p* < .05).

M, mean; SD, standard deviation; F, F-statistic; *p*, significance level; η^2^*p*, partial eta squared.

*p < .05.

### Quality of life

No significant improvement was found in perceived health over time in the different companies or by sex (see Table 3). However, pairwise comparisons showed statistically significant differences between Training and Consulting and Digital Engineering workplaces in Physical Role (*p* = .005), and between Insurance and Technology and Digital Engineering workplaces (*p* = .001). Social Functioning and Emotional Role also showed statistically significant differences between Digital Engineering workplaces and Insurance and Technology with Training and Consulting, and between Training and Consulting and both Insurance and Technology and Digital Engineering workplaces, respectively (*p* = 0.001). Statistically significant differences by sex were found in Physical Role, Social Functioning, and Emotional Role (*p* < .001).

**Table 3:** SF-36 scores by gender and workplace (pre and post-intervention).

Outcome	Gender	Company	N	Pre M	Pre SD	Post M	Post SD	F	*p*
SF-36 Physical Functioning (0–100)	Men	Training and consulting	5	96.00	4.18	95.00	6.12	.70	.41
		Insurance and technology	4	98.75	2.50	98.75	2.50		
	Women	Training and consulting	23	92.17	12.42	91.52	10.92		
		Digital engineering	1	95.00	.00	88.89	.00		
SF-36 Role Physical (0–100)	Men	Training and consulting	5	90.00	15.69	92.50	16.77	.26	.61
		Insurance and technology	4	100.00	.00	100.00	.00		
	Women	Training and consulting	23	90.49	12.62	91.85	13.38		
		Digital engineering	1	62.50	.00	50.00	.00		
SF-36 Bodily Pain (0–100)	Men	Training and consulting	5	83.20	10.64	82.60	13.92	.29	.60
		Insurance and technology	4	85.50	10.75	92.00	9.24		
	Women	Training and consulting	23	74.48	23.34	79.30	19.55		
		Digital engineering	1	74.00	.00	74.00	.00		
SF-36 General Health (0–100)	Men	Training and consulting	5	73.00	22.47	77.00	11.73	.24	.63
		Insurance and technology	4	85.75	10.31	87.00	7.07		
	Women	Training and consulting	23	67.39	25.41	71.39	22.40		
		Digital engineering	1	37.00	.00	42.00	.00		
SF-36 Vitality (0–100)	Men	Training and consulting	5	61.00	11.94	71.00	7.42	.94	.34
		Insurance and technology	4	60.00	15.81	68.75	12.50		
	Women	Training and consulting	23	54.78	11.23	61.52	8.97		
		Digital engineering	1	50.00	.00	45.00	.00		
SF-36 Social Functioning (0–100)	Men	Training and consulting	5	87.50	12.50	90.00	22.36	.96	.33
		Insurance and technology	4	93.75	12.50	93.75	12.50		
	Women	Training and consulting	23	83.70	17.85	93.48	11.84		
		Digital engineering	1	25.00	.00	37.50	.00		
SF-36 Role Emotional (0–100)	Men	Training and consulting	5	86.67	18.26	98.33	3.73	.55	.47
		Insurance and technology	4	100.00	.00	100.00	.00		
	Women	Training and consulting	23	85.51	16.33	93.12	8.58		
		Digital engineering	1	50.00	.00	50.00	.00		
SF-36 Mental Health (0–100)	Men	Training and consulting	5	64.00	18.11	74.40	10.04	1.62	.21
		Insurance and technology	4	67.00	8.87	76.00	3.27		
	Women	Training and consulting	23	62.43	13.36	69.57	9.55		
		Digital engineering	1	48.00	.00	44.00	.00		

M, mean; SD, standard deviation; F, F-statistic; *p*, significance level; η^2^*p*, partial eta squared.

### Productivity costs

Were found statistically significant differences in presenteeism-related productivity loss costs (€), with a large effect size (F₁=41.4; *p* < .001; *η*^2^ₚ=.77) (see Table 4). *post hoc* multiple comparisons revealed statistically significant differences between Insurance and Technology and Digital Engineering workplaces, as well as between men and women (*p* < .001).

**Table 4:** Absenteeism and presenteeism outcomes by gender and workplace (pre and post-intervention).

Outcome	Gender	Company	N	Pre M	Pre SD	Post M	Post SD	F	*p*	η^2^p
Absenteeism time per month (hours)	Men	Training and consulting	3	.00	.00	.00	.00	.055	.82	
		Insurance and technology	4	.00	.00	.00	.00			
	Women	Training and consulting	9	.78	2.33	.00	.00			
		Digital engineering	1	.00	.00	.00	.00			
Short-term absenteeism Productivity loss (€)	Men	Training and consulting	3	.00	.00	.00	.00	.055	.82	
		Insurance and technology	4	.00	.00	.00	.00			
	Women	Training and consulting	9	11.67	35.00	.00	.00			
		Digital engineering	1	.00	.00	.00	.00			
Absence costs during 4-week leave period (€)	Men	Training and consulting	3	.00	.00	.00	.00	.055	.82	
		Insurance and technology	4	.00	.00	.00	.00			
	Women	Training and consulting	9	75.83	227.50	.00	.00			
		Digital engineering	1	.00	.00	.00	.00			
Hours lost due to Presenteeism (part a)	Men	Training and consulting	3	.40	.53	1.00	.00	.85	.38	
		Insurance and technology	4	1.00	.00	1.00	.00			
	Women	Training and consulting	9	.89	.33	.69	.47			
		Digital engineering	1	1.00	.00	1.00	.00			
Presenteeism costs. productivity loss (€)	Men	Training and consulting	3	15.00	25.98	.00	.00	41.40	.001^**^	**.77**
		Insurance and technology	4	120.00	240.00	.00	.00			
	Women	Training and consulting	9	231.83	262.14	34.00	102.00			
		Digital engineering	1	1,800.00	.00	360.00	.00			

Bold values highlight statistically significant differences (*p* < .05).

M, mean; SD, standard deviation; F, F-statistic; *p*, significance level; η^2^*p*, partial eta squared.

**p < .01.

## Discussion

Given the limited literature on this topic, the objective of the program was to evaluate its impact from a gender perspective within the context of three real-world companies operating in different sectors. The intervention led to a significant reduction in diastolic blood pressure measurements across the participating workplaces, a decrease in health-related productivity losses due to presenteeism, and important improvements in physical fitness and cardiorespiratory recovery. Other authors in similar interventions achieved a reduction of nearly 1.79 mmHg in diastolic blood pressure, as demonstrated by the work of McEachan et al. (34), although the effects found on physical health depend on the workplace, the type of intervention, and the target population; the literature has found statistically significant improvements in this variable after five months of intervention in the service sector (35). However, our results suggest that a three-month workplace program, offered two to three times per week and combining aerobic and resistance training, active breaks, and monthly challenges, can be effective for employees in sedentary administrative roles.

Our findings indicate that the intervention may help reduce certain cardiovascular risk factors, especially among previously inactive participants, while also contributing to reductions in productivity losses associated with presenteeism. Although scientific evidence suggests that the variables examined can improve on their own through health promotion processes that enhance the physical environment and organizational structure in the workplace (36), the observed heterogeneity suggests the need to improve the methodological quality of longitudinal research, which should be theory-based and take into account the socio-labor and organizational realities of companies (45). These studies would aim to determine whether incorporating physical activity during the workday is beneficial in small and medium-sized enterprises with similar or differing labor conditions and industrial sectors (28). This approach could provide valuable insights into the role of physical activity in managing work processes, organizational behavior, and decision-making, as well as assist in developing workplace health policies aligned with corporate strategy and culture.

Also, inclusive participation of women facing barriers to regular and sustained physical activity is very important, as it can enhance adherence when supported by qualified professionals, while also promoting employment and job satisfaction (37). It seems important to conduct larger and more comprehensive studies, such as randomized controlled trials, stratified by job type and the working conditions of each company, especially considering that scientific evidence has demonstrated the effectiveness of occupational health programs on working women (38). It is recommended that physical activity be organized in group settings, led by certified Physical Education professionals, and compared against a control group that does not receive the intervention. This is especially important for adults who are not healthy (39, 40). Likewise, future research could explore the incorporation of high-intensity training with healthy participants, given that consistent preliminary evidence has been found regarding quality of life (41).

Implementing a combined program that includes challenges, active breaks, and weekly monitoring two to three days a week among companys of different sectors employees can significantly improve cardiovascular health, fitness levels, and reduce presenteeism costs. Adherence to this program may boost productivity and employee satisfaction, thereby lowering absenteeism. Future research should include sensitivity analyses based on inclusion criteria and subgroup evaluations. Overall, a structured physical exercise program appears to be a viable and effective strategy to enhance the health and well-being of workers, encouraging a more active and healthy lifestyle.

## Conclusion

The findings indicate that a supervised physical exercise program lasting three months, conducted three times per week either at the workplace or online, and tailored to the specific needs and preferences of healthy participants, may positively impact the health of administrative staff in the small and medium-sized enterprises studied. This multicomponent program, which incorporates aerobic and strength training along with monthly challenges, online workshops, and morning activation sessions, also has the potential to reduce related health and productivity costs. These results highlight the importance of implementing tailored workplace exercise interventions to promote employee well-being and organizational efficiency.

### Limitations

The small sample size increased the impact of outliers. About 20% of participants dropped out, influenced by lack of incentives, scheduling conflicts, and gender disparities. For example, the digital engineering company involved had a workforce with notably compromised health. Also, one of the companies represents 78% of the total sample, which considerably affects the comparability between groups and introduces a possible sampling bias. Due to budget and logistical constraints, it was not possible to create separate control and intervention groups, limiting internal validity.

### Future perspectives

Future research should be explore the economic impact to see if health improvements translate into organizational benefits, supporting broader implementation in SMEs. To improve retention, we recommend screening for access barriers, aligning schedules, implementing gradual withdrawal protocols, and encouraging peer support. Establishing a control group will be essential to strengthen study rigor and reliability.

## Data Availability

The original contributions presented in the study are included in the article/Supplementary Material, further inquiries can be directed to the corresponding author.
